# Altered Gene Expression in Dioxin-Like and Non-Dioxin-Like PCB Exposed Peripheral Blood Mononuclear Cells

**DOI:** 10.3390/ijerph16122090

**Published:** 2019-06-13

**Authors:** Marike M. Leijs, Lin Gan, Patrick De Boever, André Esser, Philipp M. Amann, Patrick Ziegler, Katharina Fietkau, Thomas Schettgen, Thomas Kraus, Hans F. Merk, Jens M. Baron

**Affiliations:** 1 Department of Dermatology and Allergology, RWTH Aachen University, 52074 Aachen, Germany; philipp.amann@slk-kliniken.de (P.M.A.); kfietkau@ukaachen.de (K.F.); hans.merk@post.rwth-aachen.de (H.F.M.); jbaron@ukaachen.de (J.M.B.); 2 IZKF, RWTH Aachen University, 52074 Aachen, Germany; lgan@ukaachen.de; 3 Flemish Institute for Technological Research (VITO), Health unit, 2400 Mol, Belgium; Patrick.deboever@vito.be; 4 Centre for Environmental Sciences, Hasselt University, 3590 Diepenbeek, Belgium; 5 Institute for Occupational, Social and Environmental Medicine, RWTH Aachen University, 52074 Aachen, Germany; anesser@ukaachen.de (A.E.); pziegler@ukaachen.de (P.Z.); tschettgen@ukaachen.de (T.S.); tkraus@ukaachen.de (T.K.); 6 Department of Dermatology, SLK Hospital Heilbronn, 74078 Heilbronn, Germany

**Keywords:** polychlorinated biphenyls, PBMC, gene regulation, PCB exposure, AHR, oxidative stress

## Abstract

Polychlorinated biphenyls (PCBs) are well known carcinogenic persistent environmental pollutants and endocrine disruptors. Our aim was to identify the possible dysregulation of genes in PCB exposed peripheral blood mononuclear cells (PBMCs) in order to give more insight into the differential pathophysiological effects of PCB congeners and mixtures, with an emphasis on immunological effects and oxidative stress. The PBMCs of a healthy volunteer (male, 56 years old) were exposed to a mixture of dioxin-like (DL)-PCBs (PCB 77, 81, 105, 114, 118, 123, 126, 156, 157, 167, 169, and 189, 250 µg/L resp.) or non-dioxin-like (NDL)-PCBs (PCB 28, 52, 101, 138, 153, 180, 250 µg/L resp.) or single PCB congener (no.28, 138, 153, 180, 250 µg/L resp.). After an incubation period of 24 h, a microarray gene expression screening was performed, and the results were compared to gene expression in control samples (PBMCs treated with the vehicle iso-octane). Treatment of PBMCs with the DL-PCB mixture resulted in the largest number of differentially regulated genes (181 upregulated genes >2-fold, 173 downregulated >2-fold). Treatment with the NDL-PCB mix resulted in 32 upregulated genes >2-fold and 12 downregulated genes >2-fold. A gene set enrichment analysis (GSEA) on DL-PCB treated PBMCs resulted in an upregulation of 125 gene sets and a downregulation of 76 gene sets. Predominantly downregulated gene sets were involved in immunological pathways (such as response to virus, innate immune response, defense response). An upregulation of pathways related to oxidative stress could be observed for all PCB congeners except PCB-28; the latter congener dysregulated the least number of genes. Our experiment augments the information known about immunological and cellular stress responses following DL- as well as NDL-PCB exposure and provides new information on PCB 28. Further studies should be performed to evaluate how disruption of these pathways contributes to the development of autoimmune diseases and cancer.

## 1. Introduction

Polychlorinated biphenyls (PCBs) are persistent environmental pollutants that elicit a broad spectrum of negative effects in mammals and other vertebrate species [1,2,3]. They cover a group of 209 congeners and are classified as human carcinogen group 1 by the International Agency for Research on Cancer (IARC) [4]. From the 1930s until their ban in 1977, PCBs were mainly used in electrical equipment because of their fire resistance and low electrical conductivity [5]. As chemical accidents with involving PCBs resulted in severe health effects, birth abnormalities and mortality, these compounds became notorious to the general public [6].

Individual congeners differ markedly in their chemical and toxicological properties, mainly depending on the position of the chlorine atoms on the PCB molecule [7]. Twelve congeners with co-planar structure show toxicological properties similar to dioxin and are therefore termed dioxin-like PCBs (DL-PCBs). Dioxin-like chemicals such as DL-PCBs are known to exert their effect by binding to the aryl hydrocarbon receptor (AhR), forming complexes with aryl hydrocarbon nuclear translocator (ARNT). This complex binds to dioxin responsive elements (DRE) in gene promoter regions in order to regulate the transcription of specific genes (aromatic hydrocarbon gene battery). This gene battery comprises the genes that encode the detoxifying enzymes of phase I like cytochrome P450 1A1 (CYP1A1) and 1B1 (CYP1B1) and enzymes of phase II [8].

The majority of PCB-related toxicity studies at the molecular and cellular level have focused on co-planar dioxin-like (DL) congeners [9]. DL-PCBs can act as strong immunosuppressive compounds through binding the Ah-receptor [2]. Cancer studies in rats with technical PCB mixtures suggests that the dioxin-like components are likely to be responsible for the carcinogenic response of these mixtures [10]. However, carcinogenic effects of non-dioxin-like (NDL)-PCBs have also been published [11]. Besides the genomic pathway, DL-PCBs can exert effects through the non-genomic pathway. An important role of this pathway in causing inflammatory reactions has been reported [7]. The non-dioxin-like PCBs (NDL-PCBs) exert different effects. Phenobarbital-like, estrogenic and neurotoxic effects are described, as well as effects on the thyroid regulatory pathway [3,12,13]. NDL-PCBs can induce CYP2 and CYP3 enzymes independently from AhR signaling [14,15,16]. The non-planar PCB 138, 153 and 180 are the most prevalent and persistent PCB congeners and have been reported to be the predominant congeners in humans [17]. There is evidence that NDL-PCBs interfere with the lipid metabolism and contribute to the development of obesity and metabolic diseases [18]. In addition, impairments of the immune system have been reported, including the impairment of the innate immune response in macrophages [19]. However, a human study indicated that effects on immunologic parameters are mainly correlated to DL-PCB congeners [2,20].

In 2010, high blood levels of PCBs were found in workers of a recycling company in Germany. In addition, elevated PCB plasma levels were found in the workers’ relatives, individuals working in close vicinity, persons working in the larger area, and the residents. The median plasma level for the sum of the 6 indicator PCBs (no.28, 52, 101, 138, 153, 180) of the total cohort was 3.68 µg/L (max. 236.3 µg/L). PCB 180, 153, 138 and 28 were the predominantly elevated congeners [21,22]. Toxicogenomic studies on human peripheral blood mononuclear cells (PBMCs) comparing the effects of DL-PCB mixtures to those of NDL-PCB mixtures and single PCBs, are scarce and for PCB 28 not available. Our aim was to identify differences in DL-PCB and NDL-PCB regulated genes in PBMCs as well as to identify genes regulated by PCB 180, 153, 138 and 28 in order to provide more insight into the pathophysiological effects caused by these congeners and mixtures of PCBs on gene level with an emphasis on immunological effects and on pathways responsible for oxidative stress.

## 2. Materials and Methods

This study is linked to a surveillance program on PCB exposed workers of a transformer recycling company in Germany [21].

### 2.1. Chemicals

A solution of 10 mg/L of the dioxin-like PCBs―PCB 77, 81, 105, 114, 118, 123, 126, 156, 157, 167, 169, and 189 (PCB Mix 41)―in iso-octane (Applichem, Darmstadt, Germany) and of six indicator non-dioxin-like PCBs―PCB 28, 52, 101, 138, 153, and 180 (together Mix 1) (10 mg/L in iso-octane, respectively) were obtained from Dr. Ehrenstorfer Laboratories (Augsburg, Germany). Purity for NDL-PCBs was verified in the gravimetric certificate of Dr. Ehrenstorfer and varied from 98.5%–99.0%. Further used chemicals are listed elsewhere [22].

### 2.2. PCB In Vitro Exposure

Human peripheral blood mononuclear cells (PBMCs) from a healthy donor (male, 56 years old) with no occupational- or other known PCB exposition other than the normal background exposition in Germany, were purchased from the blood bank of the RWTH University Hospital in Aachen.

PBMCs were separated from purchased single buffy coats (Department of Transfusion Medicine, University Hospital Aachen, Germany) over a Ficoll-Paque gradient (Amersham Pharmacia Biotech, Uppsala, Sweden). PBMCs were suspended in complete medium consisting of RPMI-1640 (Life Technologies, Darmstadt, Germany) enriched with 10% heat-inactivated autologous plasma and 1% L-glutamine, 1% non-essential amino acids (Life Technologies, Darmstadt, Germany) and 1% Na-pyruvate (Life Technologies, Darmstadt, Germany). The cell suspension was plated at a density of 1 × 106 cells per culture dishes (100 mm diameter).

PBMCs were treated with either way a DL- or NDL-PCB mixture or with single PCB congeners:

(1) 250 µg/L (for each congener) of the DL-PCB mix 41 (PCB 77, 81, 105, 114, 118, 123, 126, 156, 157, 167, 169, and 189) (total toxic equivalents (TEQ): 32.7 µg TEQ/L). Molarity values were as followed: For PCB 77 and 81: 856 nM; for PCB 105, 114, 118, 123 and 126: 767 nM; for PCB 156, 157, 167 and 169: 681 nM; and for PCB 189: 631nM.
(2) 250 µg/L (for each congener) of PCB mix 1 (NDL-PCB mix 1: PCB 28 (977 nM); PCB 52 (856 nM); PCB 101 (767nM); PCB 138 and 153 (681 nM); PCB 180 (631 nM).
(3) Single congener: PCB 28, 138, 153 or 180, (250 µg/L for each congener respectively: PCB 28 (977 nM); PCB 138 and 153 (681 nM); PCB 180 (631) nM).

The PBMCs were exposed for 24 h. Control cells were treated with the solvent iso-octane for 24 h, to exclude its toxicity. Afterwards, RNA preparation was initiated.

### 2.3. Microarray Analysis

Total RNA was amplified and labelled to generate complementary RNA (cRNA) using the Quick Amp Labelling (two color) kit (Agilent Technologies) according to the manufacturer’s instructions. Details are described elsewhere [23]. For quality control, the microarray data were generated from two independent exposure experiments as described above. Normalized gene expression data were loaded in GeneSpring GX 11.0.2 software (Agilent Technologies, Frankfurt am Main, Germany) and average fold changes (PCB-exposed versus vehicle control) were calculated. Alternatively, fragmentation and labeling were performed using the Affymetrix GeneChip WT Terminal Labeling kit (Affymetrix, Santa Clara, CA, USA) according to the manufacturer’s recommendations (for PCB congener no. 28, 138, 153 and 180). Each sample was hybridized to a Gene Chip Human Exon 1.0 ST array for 16 h at 45 °C.

Expression values of each probe set were determined and control samples with unstimulated probes were compared with stimulated probes using the Gene-Spring GX 11.0.2 software (Agilent Technologies, Frankfurt am Main, Germany (for the DL- and NDL-PCB mix)).

Dysregulated genes of unknown gene ID were excluded.

### 2.4. Gene Set Enrichment Analysis

To evaluate whether PCB exposure could dysregulate a set of genes from a certain pathway, we applied pre-ranked gene set enrichment analysis (GSEA) [24] of the entire ranked gene list on 201 selected gene sets of interest of the Molecular Signatures Database (MSigDB) [25] with 1000 permutations. The enrichment score (ES) and the false discovery rate (FDR q-val) was used for calculating the result of the gene set enrichment analysis. The nominal (nom) *p*-value estimates the statistical significance of the enrichment score for a single gene set. Further details of the statistical analyses can be found elsewhere [24,25].

### 2.5. RNA Isolation and qRT-PCR

RNA isolation and qRT-PCR analysis were performed as described elsewhere [26]. TaqMan experiments were carried out on an ABI Prism 7300 sequence detection system (Applied Biosystems, Weiterstadt, Germany) using Assays-on-Demand gene expression products for AhRR (Hs01005075_m1), CYP1A1 (Hs00153120_m1), CYP1B1 (Hs00164383_m1), CCL 1 (Hs00171072_m1), CCL 7 (Hs00171147_m1), CCL 8 (Hs00271615_m1), CCL 20 (Hs01011368_m1), CXCL1 (Hs00236937_m1), CXCL2 (Hs00601975_m1), interleukin (IL)-1β (Hs00174097_m1), IL-6 (Hs00985641_m1), IL-8 (Hs00174103_m1), MMP-9 (Hs00234579_m1), IL-1α (Hs00174092 _m1), TP53 (Hs01034249 _m1), according to the manufacturer’s recommendations. All measurements were performed in triplicates in separate reaction wells. Real-time polymerase chain reaction (PCR) efficiencies were determined for each primer probe set from standard curves. On the basis of the standard curves, the initial cDNA copy number was determined to calculate the fold induction values [27].

## 3. Results

### 3.1. Treatment of PBMCs with DL-PCB Mix and NDL-PCB Mix

Treatment of PBMCs with the DL-PCB mix (mix 41) led to downregulation of 173 genes (> 2-fold) and upregulation of 181 genes (>2-fold) (see Table 1 and Supplementary Materials). Highly upregulated genes included matrix metallopeptidase 9 (MMP-9), CYP1B1 and multiple interleukins (IL-8, IL-24 and IL-1beta). Highly downregulated genes included sialic acid binding Ig-like lectin 1 (SIGLECI-1), chemokines (CXCL11, CCL13, CXCL10, CCL23), leptin (LEP) and interferon related genes (IFIT, IFI27, IFI6, IFIT3, IFI44L). Treatment of PBMCs with NDL-PCB mixture resulted in a downregulation of 12 genes (>2-fold) and an upregulation of 32 genes (>2-fold, see Table 1 and Supplementary Materials). Highly downregulated genes included melanoma antigen family B6 (MAGEB6) and chemokine (C-X-C motif) ligand 5 (CXCL5). Upregulated genes included members of RAS oncogene family-like 3 (RAB, RABL3) as well as several genes involved in immunologic reactions, including chemokine (C-C motif) receptor 3 (CCR3), transcript variant 1, interleukin 1 receptor-like 1 (IL1RL1) and CXCL11.

**Figure 1:**
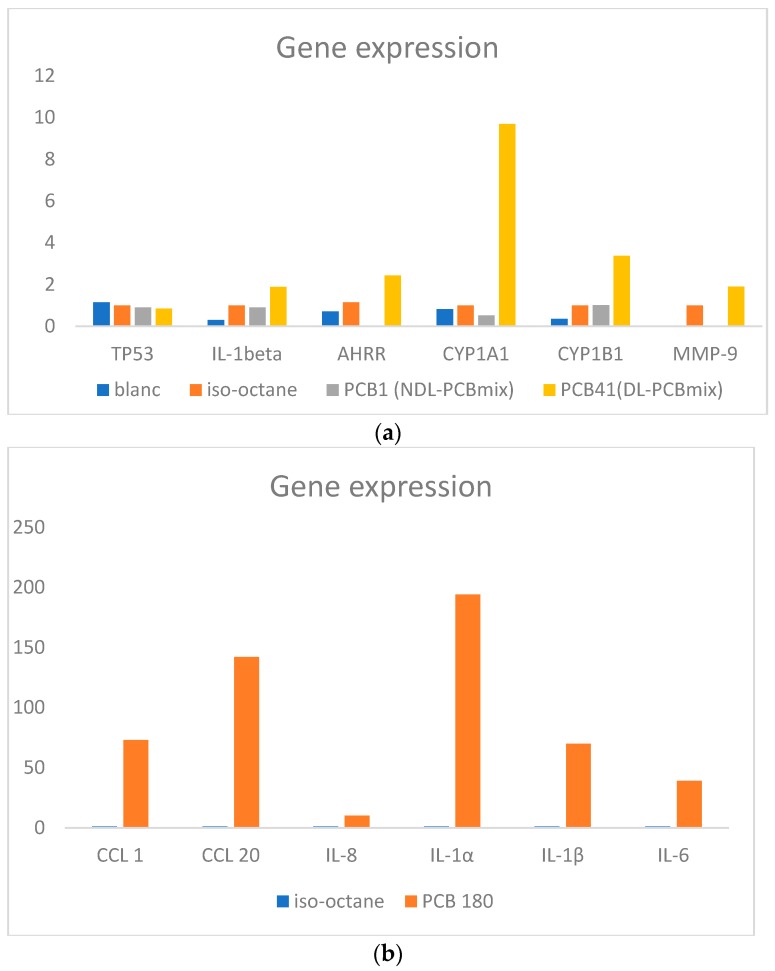
Differently regulated genes confirmed by RT-PCR: Some differently regulated genes of interest were confirmed by qRT-PCR. In the figure, their relative quantitation compared to the vehicle (iso-octane) is displayed. (**a**) shows the confirmed genes for non-dioxin-like (NDL)-PCB mix1 and dioxin-like (DL)-PCB mix41. The tumor suppressor gene (TP53) showed a slight downregulation for the DL and NDL-mixture; CYP1A1, CYP1B1, AHRR and MMP-9 were upregulated by the DL-PCBmix. (**b**) displays confirmed upregulated genes for PCB 180: CCL 1, CCL 20, IL1-α, IL1-β, IL-6. Other confirmed genes are listed in the Supplementary Materials.

A set of differentially expressed genes based on the array data were confirmed by TaqMan PCR analysis (AhRR, CYP1A1, CYP1B1, TP53, IL-8, MMP-9, CCL 8, IL-6, CCL 1, CCL 20, IL1-α, IL1-β). The main results are presented in Figure 1. While the tumor suppressor gene (TP53) showed a slight downregulation, CYP1A1, CYP1B1, AHRR and MMP-9 were upregulated by the DL-PCB mix. Further confirmed genes can be found in the Supplementary Materials.

**Figure 2:**
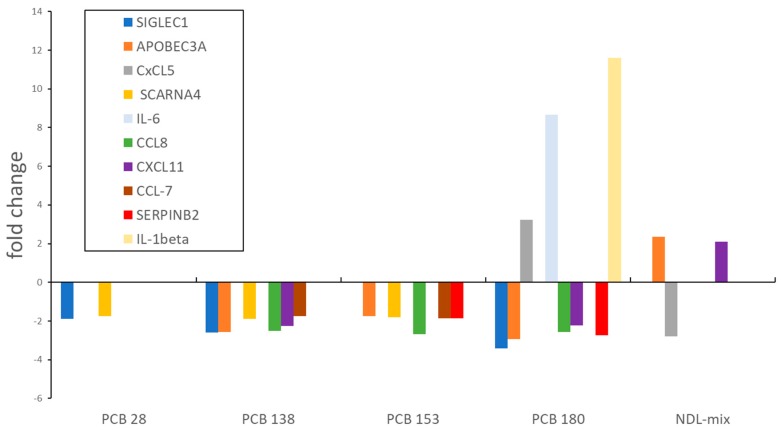
Some genes of interest commonly dysregulated by non-dioxin-like (NDL)-PCBs. Figure 2 displays a selection of commonly dysregulated genes (fold change) for the NDL- PCBs (congener 28, 138, 153, 180, as well as the NDL-PBC mixture (NDL-mix). Some of these genes (IL-6, CCL-7, IL1-α, IL1-β) were confirmed by qRT-PCR. The gene CXCL11 was (-4.32 fold) downregulated in PBMCs stimulated with DL-PCBs and upregulated with NDL-PCBs (2.1 fold) mix. PCB 180 exposed PBMCs showed a downregulation of CXCL11 of−2.2 fold.

PBMCs were treated with the NDL-PCB 28, 138, 153 or 180 and were related to vehicle (iso-octane) treated control. The pattern and number of upregulated genes were strongly dependent on the type of congener. PCB 180 was the congener with the most dysregulated genes (downregulation of 27 genes, upregulation of 68 genes >2-fold) such as CCL 8 and 20, CXCL1, 2, 5, and 11 as well as IL-6 (see Figure 2).

Fewer genes were differentially expressed following treatment with PCB 138: a downregulation of 20 genes (> 2-fold) of known function. PCB 153 triggered an upregulation of only 1 (> 2-fold) gene and a downregulation of 3 genes (> 2-fold). PCB 28 downregulated of 1 gene (> 2-fold).

### 3.2. Different Expression Patterns in Different Congeners/Mixtures

**Figure 3:**
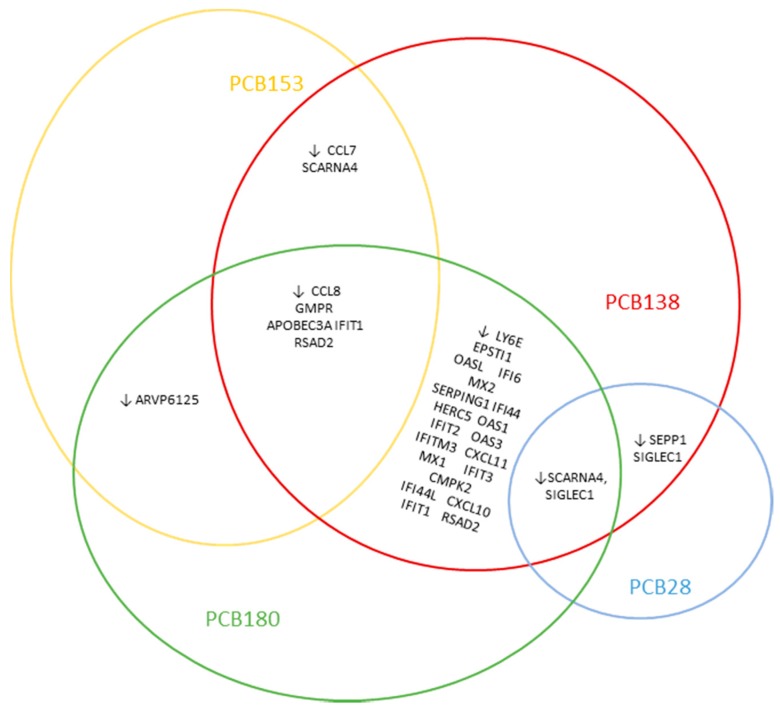
Commonly downregulated genes by single PCB congeners. PCB 180 and 138 contained most commonly downregulated genes. No genes were commonly upregulated.

We examined which genes were up- or downregulated in common by single PCB congeners or mixtures (see Table 2 and Figure 3): PCB 153 and 138 downregulated 7 similar genes: CCL7, SCARNA4, CCL8, GMPR, APOBEC3A, IFIT1 and RSAD2. PCB 138 had the most downregulated genes in common with PCB 180: LY6E, EPSTI1, OASL, IFI6, MX2, SERPING1, IFI44, HERC5, OAS1, IFIT2, OAS3, IFITM3, CXCL11, MX1, IFIT3, CMPK2, CCL8, GMPR, IFI44L, APOBEC3A, SIGLEC1, CXCL10, IFIT1 and RSAD2 were downregulated by both congeners. PCB 138 and PCB 28 downregulated 3 common genes: SCARNA4, SEPP1 and SIGLEC1. PCB 153 and 180 commonly downregulated 6 genes: GMPR, APOBEC3A, ARVP6125, IFIT1, RSAD2 and CCL8. No genes were commonly upregulated by the single PCB congeners.

Table 2 displays the number of commonly up- or downregulated genes of PBMCs treated with the PCB mixtures (NDL- or DL) or single congeners (28, 138, 153, 180). PCB 138 treated PBMCs, commonly downregulated 24 genes with PCB 180. PCB 180 treated PBMCs commonly downregulated 24 genes with the DL-PCB mix.

### 3.3. Gene Set Enrichment Analysis

Besides the individual analysis of differently expressed genes, we performed a GSEA to see whether genes representing certain pathways were altered by PCBs. Stimulation of PBMCs with the DL-PCB mix resulted in upregulation of 125 gene-sets (from which seven gene sets *p* < 1% and 25 *p* < 5%) and 76 Gene-sets were downregulated (from which 16 gene sets *p* < 1% and 26 *p* > 5%). GO (gene ontology) GSEA revealed a disruption of multiple signaling pathways (GO-Enrichment score) related to inflammation. Top scoring downregulated pathways included are given in Table 3.

Top positively regulated pathways included: Go-positive-regulation-of-multicellular-organismal-process, Go-embryo-development, Go-locomotion and Go-cellular-response-to-stress (see Table 4).

For single NDL-PCB congeners, we found fewer differentially regulated genes. Therefore, we included also >1.7 fold change differential regulated genes. For PCB-28, altered regulated genes were in a smaller amount (0 upregulated- and 5 downregulated genes of known gene ID). As a result of that, we did not find any enriched pathways. Stimulation with PCB 180 resulted in 96 upregulated gene sets and 9 downregulated gene sets. Top scoring gene sets were: Go-cell-chemotaxis, Go-cytokine-activity, Go-cell-motility, Go-defense-response-to-other-organism, Go-immune effector-process, Go-Inflammatory response, Go-innate- Immune response, Go-negative-relation of cell proliferation, Positive regulation of MAPK cascade, Go-response to virus.

Stimulation with PCB 138 resulted in a downregulation of 14 gene-sets (6 gene sets had a *p* < 5%). Downregulated gene-sets included: Go-response-to-cytokine, Go-cytokine-mediated-signalling, Go-defence-response. The total number of dysregulated gene sets for both PCB mixtures (NDL- and DL) as well as PCB 28, 153, 138, 18 are reported in Table 5.

Table 5 displays the total number of dysregulated gene sets for both PCB mixtures (NDL- and DL) as well as PCB 28, 153, 138, 180.

Oxidative stress related gene sets were upregulated for DL- (8 gene sets) and NDL-PCB (4 gene sets) mixtures, as well as for PCB 180 (10 gene sets upregulated), PCB 153 (1 gene set) and PCB 138 (2 gene sets) treated PBMCs. The statistically significantly upregulated oxidative stress related pathways of PCB 180 are summarized in Table 6.

Table 6 displays statistically significantly upregulated oxidative stress related pathways of PCB 180.

## 4. Discussion

Microarray analysis showed that DL- and NDL-PCB exposure of PBMCs causes differential gene expression. Although the number and type of genes dysregulated differed significantly for each congener or mixture, commonly dysregulated genes/pathways were also identified (Table 2, Figure 1a, Figure 2 and Figure 3). By using GSEA, most differently regulated pathways were found for DL-PCBs. For PCB-28, no differently regulated pathways were found.

We found an upregulation of matrix-matallopeptidases like MMP-2, MMP-7, and MMP-9 in DL-PCB treated PBMCs (see Supplementary Materials, Figure 1a). This is in line with another study on urothelial carcinoma cells, which found that AhR ligands like 2,3,7,8-tetrachlorodibenzo-p-dioxin (TCDD) upregulate expression of matrix metalloproteinases in vitro. It was hypothesized that the induction of extracellular matrix components such as the matrix metalloproteinases may contribute to dioxin-induced cancer invasion and metastasis [28,29].

AhRR serves as a negative feedback regulator of AhR induction and represses its transcription activity by competing with this transcription factor for heterodimer formation with ARNT [30]. Consistently, we found AhRR upregulation in DL-PCB treated PBMCs (see Figure 1a). Most likely via the AhR pathway, DL-PCBs are able to induce an inflammatory response [31]. In this respect, we observed a significant dysregulation of inflammatory mediators such as IL-1β, CXCL11, CCL13, CXCL10, CCL23 and interferon-related genes IFIT, IFI27, IFI6, IFIT3, IFI44 for DL-PCB treated PBMCs at mRNA level. However, also non-AhR pathways play an important role in the inflammatory action of dioxin-like compounds [32]. Some of these disrupted inflammatory mediators, like CXCL11, CXCL20, IL-6, IL-1β, are involved in the pathogenesis of autoimmune disorders and play a potential role in tumor progression and metastasis [33,34,35,36,37]. CCL20 plays an important role in the pathogenesis of autoimmune neurological diseases [34]. Studies have shown that IL-1 cytokines are tumor promoting, mainly due to the fact that they cause a chronic inflammation [38]. Interestingly, an upregulation of IL-1β was seen in vivo in (chronically) PCB exposed workers [39]. In this study, PCB-180 was one of the main elevated congeners in the workers compared to levels of the normal population.

Effects due to NDL-PCBs involve multiple unrelated mechanisms of action [13]. Because of structural differences, they can modulate gene-expression through different pathways. In our experiment, CXCL11 and APOBEC3A were upregulated by the NDL-PCB mix while downregulated by PCB 180 and 138 (see Figure 2). Other in vitro gene expression studies also showed different regulatory effects while comparing DL- and NDL-PCB exposure [40,41]. However, in these studies other cell lines were used (human liver line). One other study on human PBMCs found differently regulated pathways for DL- and NDL-PCBs stimulated PBMCs as well. In addition, this study showed gender-specific differences [42].

Importantly, animal studies have shown that NDL-PCBs such as PCB 153 can antagonize or increase the effects of dioxin-like compounds [43,44]. In addition, AhR and non-AhR effects are difficult to separate on gene-level. One study found that DDE and PCB 153 up-regulated expression of AhR mRNA independent of direct AhR activation [45]. It was suggested that the induction of AhR expression was induced by NF-κB which promotes an inflammatory loop, via pro-inflammatory cytokines expression as interleukin (IL)-1 β and TNFα [46]. We did not find an AhR activation for the NDL-PCBs, however an upregulation of the interleukine-1 receptor-like 1 (IL1RL1) was seen in PBMCs after exposure to the NDL-mix.

PCB 153 is a di-ortho-substituted non-planar PCB and is present at the highest concentrations in human samples on a molar basis. In our experiment, gene expression pattern of the NDL-PCB congeners PCB 138 and 153 was partly overlapping in contrast to previous work of Ghosh et al. (2011). They found that PCB 138 and PCB 153 act differently in terms of differential gene expression, and towards disease and developmental processes in significantly different pathways [47]. In this study however, the PBMCs were exposed for 48 h (in our study 24 h) and lower concentrations of PCB (138 and 153) were used. This could be an explanation for the fact that other differently expressed genes were observed our study compared to the above-mentioned one.

Differentially expressed genes were found in a study on human B lymphoblastoid cells exposed to PCB-153. One of the differentially expressed genes was CCL-20, which was suggested as a possible biomarker. In our experiment we found for PCB 153 no differential regulation for CCL-20, however we found a downregulation for CCL-7, CCL-8 and CXCL-5. In another study on human hepatic cell lines, treatment with PCB 153 resulted in upregulation of pathways related with oxidative stress [41], as seen in our experiment.

Of all tested NDL-PCB congeners, most genes were differentially regulated by PCB 180, a di-ortho PCB congener with 7 chloratoms. In addition, PCB-180 had, from all PCB congeners, most genes in common with the DL-PCB mix (24 commonly downregulated genes). We assume that different NDL-PCB congeners mediate gene regulatory effects differently via independent downstream pathways. Dysregulation of genes from PCB 180 treated PBMCs could be induced by indirect AhR activation [46]. Effects through the non-classical pathway have been identified to play an important role in the inflammatory action of dioxin-like- and non-dioxin-like compounds [7], as well as for other processes like insulin secretion, fat storage, liver damage and tumorigenesis [8]. The estimated biological half-life of PCB 180 is, like some of the other congeners, relatively long (11.5 years) and could thus alter signaling pathways in vivo over a longer period of time [48].

Just a few toxicogenomic studies on PBMCs treated with single NDL-PCB congeners have been performed. One study on rats found other differential regulated pathways than the ones found in our study. Particularly enriched gene lists in this study included genes of the nervous system development and function ontology. Besides that, enriched pathways included lipid metabolism, molecular transport, small molecule biochemistry, and cell signaling and proliferation [49]. We have to keep in mind, that in this study PBMCs of other species were used and the time of the exposure was in utero. One study on a PCB exposed cohort showed alterations in pathways involved in cell-to-cell signaling and interaction, cellular movement, cell signaling, molecular transport, and vitamin and mineral metabolism in PBMCs of high PCB-exposed children. This is suggested to play a role in the possible development of diseases and disorders, including cardiovascular disease and cancer [9].

There is abundant evidence that NDL-PCB congeners have toxic effects on various organ systems [13]. One of the most prominent upregulated genes in the NDL-PCB congeners was APOBEC3A, which is involved in oncogenesis of a sizeable proportion of human cancers. APOBEC3A/B-induced mutations were described for many cancer types in previous study [50]. In addition, APOBEC3A is shown to be upregulated following HPV16 infection and increases the chance of viral DNA integration. Thereby APOBEC3A might play a role in HPV-induced carcinogenesis [51].

Several NDL-PCB congeners are metabolized to hydroxy-PCBs and/or methylsulfonyl-PCBs. Some of these metabolites may contribute to endocrine disruptive effects as seen after PCB exposure [10]. The congener PCB 28 has been shown to reduce telomerase activity in skin cells [52] and suppresses apoptosis in rat hepatocytes, a mechanism linked to promotion of carcinogenesis [53]. PCB 28 was shown to affect nearly 600 genes in vivo in a mouse experiment [52,54]. Interestingly, in our in vitro experiments only five genes were differently regulated (>1.7 fold) in PCB 28-treated PBMCs. These results indicate that PCB 28 itself seems not to possess biological activity in human PBMCs. However, its metabolites in vivo could induce gene regulatory effects. In addition, all PCB congeners except for PCB-28 dysregulated oxidative stress related pathways. For PCB-180, the largest number of pathways involved in oxidative stress were upregulated. One of the enriched pathways was the MAPK related pathway. Mitogen-activated protein kinase (MAPK) controls fundamental cellular processes such as growth, proliferation, differentiation, migration and apoptosis. Abnormalities in mitogen-activated protein kinase (MAPK) signaling affects all these processes, and plays a critical role in the development and progression of cancer [55].

These analyses provide a comprehensive description of the gene expression responses which occur in human PBMCs after exposure to DL- and NDL-PCBs. We found differently regulated pathways including pathways involved in immunological response and oxidative stress. We have to keep in mind, that in real live, exposure occurs to mixtures of multiple compounds and as well as its metabolites. Further study on PBMCs of multiple individuals should be performed to filter out age- and gender-related differences.

To our knowledge, this is the first study that evaluates effects of PCB 28 on human PBMCs and compares its pattern of gene dysregulation with other NDL-PCB congeners as well as with NDL- and DL-mixtures. Further studies are recommended to examine possible effects of its metabolites.

## 5. Conclusions

In conclusion, we found an altered regulation of genes involved in important biological pathways such as immunological pathways and cellular stress. From all of the NDL-PCBs, the treatment of PBMCs with PCB 180 resulted in most of the differentially regulated genes. All of the PCB congeners except for PCB 28 upregulated pathways involved in oxidative stress. Effects related to PCB-28 exposure in vivo might be related to its metabolites, since no differentially regulated pathways were found. Further study should be performed to examine the role of this altered gene regulation in the development of cancer and autoimmune diseases.

## Figures and Tables

**Table 1:** Dysregulated genes in PBMCs after treatment to NDL- or DL-PCB mixtures or single congener (28, 138, 153, 180).

PCB No./Mixture	Upregulated Genes (>2 fold)	Downregulated Genes (>2 fold)
DL-PCB (77, 81, 105, 114, 118, 123, 126, 156, 157, 167, 169, and 189)	181	173
NDL-PCB (28, 138, 153, 180)	32	12
PCB-28	0	1
PCB-153	1	3
PCB-138	0	20
PCB-180	68	27

**Table 2:** Number of common differentially expressed genes.

	NDL-pcbmix. down	NDL-pcbmix. up	pcb138. down	pcb138. up	pcb153. down	pcb153. up	pcb180. down	pcb180. up	pcb28. down	pcb28. up	DL-pcbmix. down	DL-pcbmix. up
NDL-pcbmix.down		0	0	0	1	0	0	1	0	0	0	0
NDL-pcbmix.up			2	0	1	0	2	2	0	0	4	4
pcb138.down				0	7	0	24	0	3	0	23	0
pcb138.up					0	0	0	0	0	0	0	0
pcb153.down						0	6	2	1	0	5	1
pcb153.up							0	0	0	0	0	0
pcb180.down								0	1	0	24	1
pcb180.up									0	0	0	10
pcb28.down										0	2	0
pcb28.up											0	0
DL-pcbmix.down												0
DL-pcbmix.up												

**Table 3:** Downregulated gene sets (GSEA) of PBMCs treated with the DL-PCB mixture.

GSEA Term Name	ES	FDR q-val
GO_RESPONSE_TO_VIRUS	−0.67	0.000
GO_INNATE_IMMUNE_RESPONSE	−0.51	0.000
GO_DEFENSE_RESPONSE	−0.42	0.000
GO_CYTOKINE_MEDIATED_SIGNALING_PATHWAY	−0.48	0.000
GO_DEFENSE_RESPONSE_TO_VIRUS	−0.65	0.000
GO_IMMUNE_EFFECTOR_PROCESS	−0.58	0.000
GO_IMMUNE_RESPONSE	−0.43	0.000
GO_IMMUNE_SYSTEM_PROCESS	−0.38	0.000
GO_RESPONSE_TO_TYPE_I_INTERFERON	−0.65	0.000
GO_CELLULAR_RESPONSE_TO_CYTOKINE_STIMULUS	−0.40	0.000
GO_DEFENSE_RESPONSE_TO_OTHER_ORGANISM	−0.52	0.001
GO_RESPONSE_TO_BIOTIC_STIMULUS	−0.42	0.002
GO_RESPONSE_TO_CYTOKINE	−0.37	0.002
GO_RESPONSE_TO_EXTERNAL_STIMULUS	−0.34	0.003
GO_POSITIVE_REGULATION_OF_IMMUNE_SYSTEM_PROCESS	−0.35	0.020
GO_CELLULAR_RESPONSE_TO_ORGANIC_SUBSTANCE	−0.28	0.020
GO_REGULATION_OF_IMMUNE_SYSTEM_PROCESS	−0.30	0.044
GO_REGULATION_OF_IMMUNE_RESPONSE	−0.39	0.062
GO_CHEMICAL_HOMEOSTASIS	−0.37	0.062
GO_VESICLE_MEDIATED_TRANSPORT	−0.33	0.075
GO_CELL_CELL_SIGNALING	−0.37	0.076
GO_REGULATION_OF_DEFENSE_RESPONSE	−0.34	0.083
GO_POSITIVE_REGULATION_OF_IMMUNE_RESPONSE	−0.41	0.079
GO_EXTRACELLULAR_SPACE	−0.24	0.143
GO_LEUKOCYTE_MIGRATION	−0.38	0.144
GO_REGULATION_OF_CELL_PROLIFERATION	−0.27	0.151
GO_CELLULAR_CATABOLIC_PROCESS	−0.34	0.152

The nominal *p*-value (*p*-nom) < 0.05 for all displayed gene sets. ES = enrichment score, FDR = false discovery rate.

**Table 4:** Upregulated gene sets (GSEA) of PBMCs treated with the DL-PCB mixture.

GSEA Term Name	ES	FDR q-val
GO_POSITIVE_REGULATION_OF_MULTICELLULAR_ORGANISMAL_PROCESS	0.35	0.000
GO_INTRACELLULAR_SIGNAL_TRANSDUCTION	0.44	0.007
GO_EMBRYO_DEVELOPMENT	0.48	0.004
GO_REGULATION_OF_CELL_DEVELOPMENT	0.47	0.007
GO_REGULATION_OF_CELLULAR_COMPONENT_MOVEMENT	0.38	0.007
GO_LOCOMOTION	0.32	0.010
GO_CELL_SURFACE	0.40	0.008
GO_CELL_MOTILITY	0.33	0.020
GO_REGULATION_OF_MULTICELLULAR_ORGANISMAL_DEVELOPMENT	0.32	0.009
GO_RESPONSE_TO_WOUNDING	0.40	0.013
GO_POSITIVE_REGULATION_OF_DEVELOPMENTAL_PROCESS	0.32	0.029
GO_EXTRACELLULAR_STRUCTURE_ORGANIZATION	0.40	0.016
GO_MOVEMENT_OF_CELL_OR_SUBCELLULAR_COMPONENT	0.31	0.026
GO_NEGATIVE_REGULATION_OF_CELL_COMMUNICATION	0.35	0.030
GO_REGULATION_OF_ANATOMICAL_STRUCTURE_MORPHOGENESIS	0.33	0.032
GO_CELLULAR_RESPONSE_TO_STRESS	0.39	0.026
GO_NEGATIVE_REGULATION_OF_RESPONSE_TO_STIMULUS	0.30	0.034
GO_INTRINSIC_COMPONENT_OF_PLASMA_MEMBRANE	0.28	0.030
GO_POSITIVE_REGULATION_OF_CELLULAR_COMPONENT_ORGANIZATION	0.37	0.042
GO_MULTI_ORGANISM_REPRODUCTIVE_PROCESS	0.34	0.050

nominal *p*-value (*p*-nom) < 0.05 for all displayed gene sets. ES = enrichment score, FDR = false discovery rate.

**Table 5:** Upregulated gene sets (GSEA).

PCB Congener or Mixture	Downregulated Gene-Sets	Upregulated Gene-Sets
DL-PCB mixture	125	76
NDL-PCB mixture	0	4
PCB 28	0	0
PCB 138	14	3
PCB 153	0	1
PCB 180	9	96

**Table 6:** Upregulation of oxidative stress related pathways in PCB180 treated PBMCs.

GSEA Pathway	ES	FDR q-val
GO_RESPONSE_TO_OXIDATIVE_STRESS	0.858	0.023
GO_POSITIVE_REGULATION_OF_STRESS_ ACTIVATED_PROTEIN_KINASE_SIGNALING_CASCADE	0.945	0.023
GO_REGULATION_OF_STRESS_ ACTIVATED_PROTEIN_KINASE_SIGNALING_CASCADE	0.945	0.023
GO_CELLULAR_RESPONSE_TO_STRESS	0.763	0.017
GO_REGULATION_OF_CELLULAR_RESPONSE_TO_STRESS	0.860	0.021

## Supplementary Materials

The following are available online at https://www.mdpi.com/1660-4601/16/12/2090/s1, Supplementary File 1.
