# Supplementary material for: Altered Gene Expression in Dioxin-Like and Non-Dioxin-Like PCB Exposed Peripheral Blood Mononuclear Cells
Source: Int J Environ Res Public Health. 2019 Jun 13;16(12):2090. doi: 10.3390/ijerph16122090 (PMC6617415; doi:10.3390/ijerph16122090)
Supplement: Supplementary file 1 [file ijerph-16-02090-s001.zip › ijerph-494523-supplementary.pptx]

## Slide 1
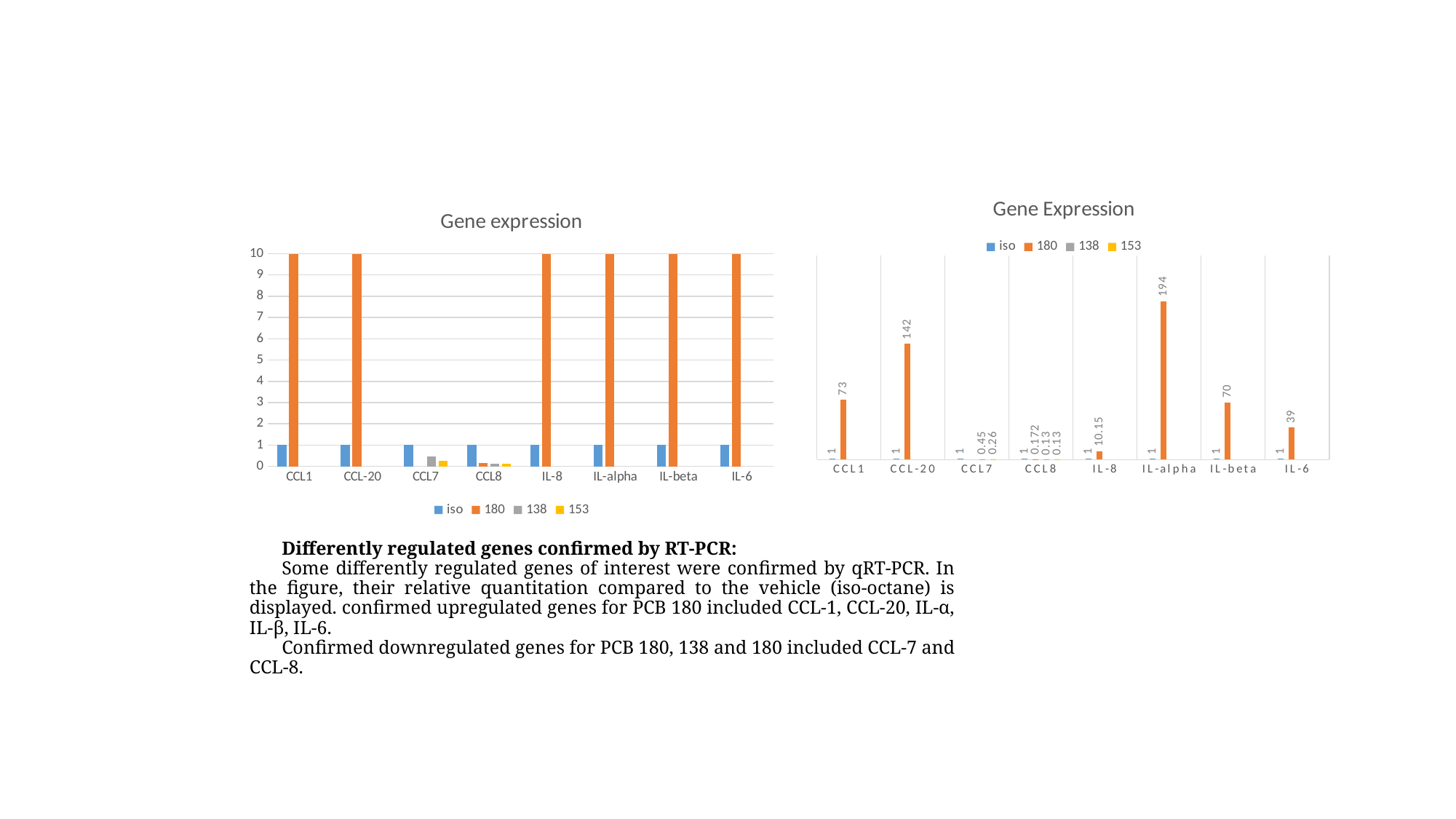

### Chart: Gene Expression
| Category | iso | 180 | 138 | 153 |
|---|---|---|---|---|
| CCL1 | 1.0 | 73.0 | None | None |
| CCL-20 | 1.0 | 142.0 | None | None |
| CCL7 | 1.0 | None | 0.45 | 0.26 |
| CCL8 | 1.0 | 0.172 | 0.13 | 0.13 |
| IL-8 | 1.0 | 10.15 | None | None |
| IL-alpha | 1.0 | 194.0 | None | None |
| IL-beta | 1.0 | 70.0 | None | None |
| IL-6 | 1.0 | 39.0 | None | None |
### Chart: Gene expression
| Category | iso | 180 | 138 | 153 |
|---|---|---|---|---|
| CCL1 | 1.0 | 73.0 | None | None |
| CCL-20 | 1.0 | 142.0 | None | None |
| CCL7 | 1.0 | None | 0.45 | 0.26 |
| CCL8 | 1.0 | 0.172 | 0.13 | 0.13 |
| IL-8 | 1.0 | 10.15 | None | None |
| IL-alpha | 1.0 | 194.0 | None | None |
| IL-beta | 1.0 | 70.0 | None | None |
| IL-6 | 1.0 | 39.0 | None | None |Differently regulated genes confirmed by RT-PCR:
Some differently regulated genes of interest were confirmed by qRT-PCR. In the figure, their relative quantitation compared to the vehicle (iso-octane) is displayed. confirmed upregulated genes for PCB 180 included CCL-1, CCL-20, IL-α, IL-β, IL-6.
Confirmed downregulated genes for PCB 180, 138 and 180 included CCL-7 and CCL-8.
